# Strengths and weaknesses in the implementation of the IHR (2005) in low GNI countries

**DOI:** 10.1093/eurpub/ckac131.577

**Published:** 2022-10-25

**Authors:** M Vasic, V Karadzic, D Jovanovic, B Kilibarda, V Jovanovic

**Affiliations:** 1 Institute of Public Health of Serbia, Belgrade, Serbia; 2 Faculty of Dentistry, Pancevo, Serbia

## Abstract

**Background:**

The International Health Regulations (IHR) 2005 are an international legal instrument adopted by World Health Organization General Assembly to strengthen global health security and empower the countries to prevent and respond to public health threats.

**Methods:**

This study was done within the Joint Action on Strengthened International Health Regulations and Preparedness in the EU (SHARP JA) with the aim to assess the strengths and weaknesses in preparedness and IHR implementation in order to develop and improve countries’ IHR capacities. Desk-based review and analysis of available data from the States Parties Annual Report tool (SPAR) from 2019 were done. Data (for 13 IHR capacities with 24 indicators) were analyzed for 15 countries participating in SHARP JA with gross national income (GNI) less than 90% of the EU average.

**Results:**

The overall capacity for IHR implementation in low GNI countries is 69%, ranging from 33% in Bosnia and Herzegovina to 84% in Spain. The highest capacities are recognized in the area of Legislation and financing - 78%, IHR Coordination & NFP Functions - 77%, Food Safety - 76%, and Health service provision - 76%. The weak areas with the need for further improvement are recorded in the Points of entry (PoE) - 51%, Risk Communication - 52%, Chemical events - 60%, National health emergency framework - 65%, Surveillance - 66%, and Human Resources - 67%. The overall IHR capacity of low GNI countries is lower compared to the SHARP JA countries and the European average (76% and 73% respectively).

**Conclusions:**

The results have shown the strongest and the weakest points of the IHR implementation in low GNI countries. This indicates the need for capacity building and further development in identified areas (Points of entry, Risk Communication, Chemical events, National health emergency framework, Surveillance, and Human Resources).

**Key messages:**

• The assessment of the strengths and weaknesses in preparedness and IHR implementation is the key prerequisite for identifying countries’ needs for their capacity enhancement.

• Special attention should be given to the strengthening low GNI countries’ capacities.

